# Seeking Abortion Care Across State Lines After the *Dobbs* Decision

**DOI:** 10.1001/jamanetworkopen.2026.1068

**Published:** 2026-03-09

**Authors:** Alia Cornell, Brianna Keefe-Oates, Olivia Thornton, Jennifer Fortin, Andrea Gallegos, Elizabeth Janiak

**Affiliations:** 1 Department of Obstetrics and Gynecology, Brigham and Women’s Hospital, Boston, Massachusetts; 2 now with Kaiser Permanente Bernard J. Tyson School of Medicine, Pasadena, California; 3 The Roux Institute at Northeastern University, Portland, Maine; 4 Planned Parenthood League of Massachusetts, Boston; 5 now with Planned Parenthood Rocky Mountains, Denver, Colorado; 6 Alamo Women’s Clinic of Illinois, Carbondale; 7 Harvard Medical School, Boston, Massachusetts; 8 Department of Social and Behavioral Sciences, Harvard T.H. Chan School of Public Health, Boston, Massachusetts

## Abstract

**Importance:**

State-level abortion restrictions in the US enforced after the June 2022 *Dobbs v Jackson Women’s Health Organization* Supreme Court decision have worsened geographic, racial, and socioeconomic inequities in abortion access, warranting investigation of experiences for individuals who travel out of state to seek abortion care.

**Objectives:**

To highlight the obstacles abortion seekers must overcome to obtain care, to identify common barriers and facilitators of interstate abortion travel, and to offer recommendations for abortion organizations, policymakers, and advocates to expand access for individuals living in states with abortion bans.

**Design, Setting, and Participants:**

This qualitative study analyzed data obtained from out-of-state participants collected as part of a larger cross-sectional, mixed-methods study. All English- and Spanish-speaking patients from out of state who were at least 16 years of age and medically cleared for research attending 2 abortion clinics in Illinois between September and November 2023 were eligible.

**Main Outcomes and Measures:**

Participants completed a self-administered survey and a semistructured interview. Directed content analysis was used to identify emergent and crosscutting themes.

**Results:**

In total, 33 individuals (all self-identified as female or a woman) completed the survey and interview. Most participants were between 20 and 24 years of age (n = 12 [36%]) or 30 years or older (n = 12 [36%]), and from southern US states with a total abortion ban (n = 25 [76%]). From the time participants decided to terminate their pregnancy to the day of the abortion appointment, they experienced a median of 14.0 days of delay, with a mean (SD) of 29.6 (36.5) days of delay. Patient journeys of seeking abortion were categorized into 3 phases: information gathering, planning finances and travel, and traveling and completing the appointment. Participants reflected on how restrictive policies in their home states deepened abortion stigma and stifled access to accurate information. Most participants encountered their longest delays in the information-gathering phase, as they had to find legitimate clinics, often without any referrals, and navigate the complex web of laws between states. When patients had personal financial security, strong social support, or were in geographic proximity to a state in which abortion was legal, their journeys were facilitated.

**Conclusions and Relevance:**

In this cross-sectional qualitative study of participants traveling across state lines for abortion, the policy landscape and abortion stigma of the home state, information and resource availability, and interpersonal support shaped their journeys in the post-*Dobbs* landscape. To improve cross-state abortion access for individuals in states with abortion restrictions or bans, policy change should be coupled with increased visibility of accurate information, charitable funding, and abortion stigma reduction. Abortion support organizations and the public should continue to enable access through social support services, such as volunteer drivers and abortion doulas.

## Key Points

**Question:**

What are the experiences of people traveling for abortion care from states with abortion restrictions or bans to Illinois, where abortion is legal, after *Dobbs*?

**Findings:**

This qualitative study using semistructured interview data from 33 participants revealed extraneous and heterogeneous paths to obtaining abortion care after *Dobbs.* Participants encountered long delays in information-gathering, searching for clinics, and navigating complex laws between states, with common facilitators and barriers to obtaining care including an individual’s financial, geographic, and social circumstances.

**Meaning:**

Findings suggest that people in states with abortion bans face limitations to obtaining abortion care out of state and should be supported through policy change, visible information and resources, and charitable and interpersonal social support systems.

## Teaser

This qualitative study investigates the experiences of people traveling from states with abortion restrictions or bans after the US Supreme Court *Dobbs v Jackson Women’s Health Organization* decision to identify common barriers and facilitators of interstate travel for abortion and obstacles to abortion care.

## Introduction

The social and economic well-being of individuals, families, and communities depends on full-spectrum reproductive health care, including induced abortion.^1,2,3^ Being denied an abortion increases the risk of pregnancy-related illness and death, contributes to adverse mental health conditions, and exacerbates suboptimal developmental outcomes for children.^3,4^ Safe and timely abortion access is associated with economic and social growth, making it a population-level public health concern when access is limited.^3^

Between 1973 and 2022, *Roe v Wade* (1973) and *Planned Parenthood v Casey* (1992) protected the right to abortion in the US.^5,6,7^ However, abortion access has always been constrained by societal dynamics of power and oppression, including structural and interpersonal racism, ableism, and misogyny, as well as geographic location.^8,9^ During broad legality, states still imposed waiting periods, clinic regulations that reduced health care professional numbers, and gestational limits, creating marked state-level disparities.^7,10^ Given these barriers, some populations have always had to travel for abortion care.^11,12,13^

Even when legal, abortion has rarely been covered by insurance in the US, and the out-of-pocket costs are prohibitively expensive.^14,15,16,17^ In 2023, it was estimated that on average, patients paid $563 for a medication abortion (up to approximately 11 weeks), $650 for a first-trimester procedural abortion (up to 14 weeks), and $1000 for a second-trimester procedural abortion (≥14 weeks).^18^ Given that 41% of people obtaining abortions had an income below the federal poverty level, these costs are prohibitive.^19^ One study found these costs to be catastrophic for all households earning up to their state’s median monthly income in all 50 states and Washington, DC.^16^

When patients are required to travel, they incur additional costs, such as gas, plane tickets, accommodation, lost wages, and food, which skyrocket the overall cost of an abortion.^20^ Planning for these costs can lead to delays in receiving care or impede the ability to receive care altogether.^13,21,22,23^ Charitable funds may offset the cost of the procedure or assist with travel; however, funding availability can vary widely based on location and is estimated to cover only between 10% and 20% of all abortions in the country.^24^ The financial burdens of an abortion may fall disproportionately on persons most marginalized in the health care system, propagating unjust cycles of health, social, and economic strain on these groups.^25,26,27,28,29^

In June 2022, the Supreme Court’s decision in *Dobbs v Jackson Women’s Health Organization* overturned *Roe v Wade* and *Planned Parenthood v Casey*.^5,6,30^ The Supreme Court ruled that the US Constitution does not protect the right to abortion and gave states full authority to regulate abortion legality.^30^ As a result, many states were able to enforce abortion restrictions that federal court injunctions had previously blocked.^30,31^ This created an even more fragmented patchwork of abortion laws across the country, affecting an estimated 25 million people of reproductive age.^32^

**Figure 1.**
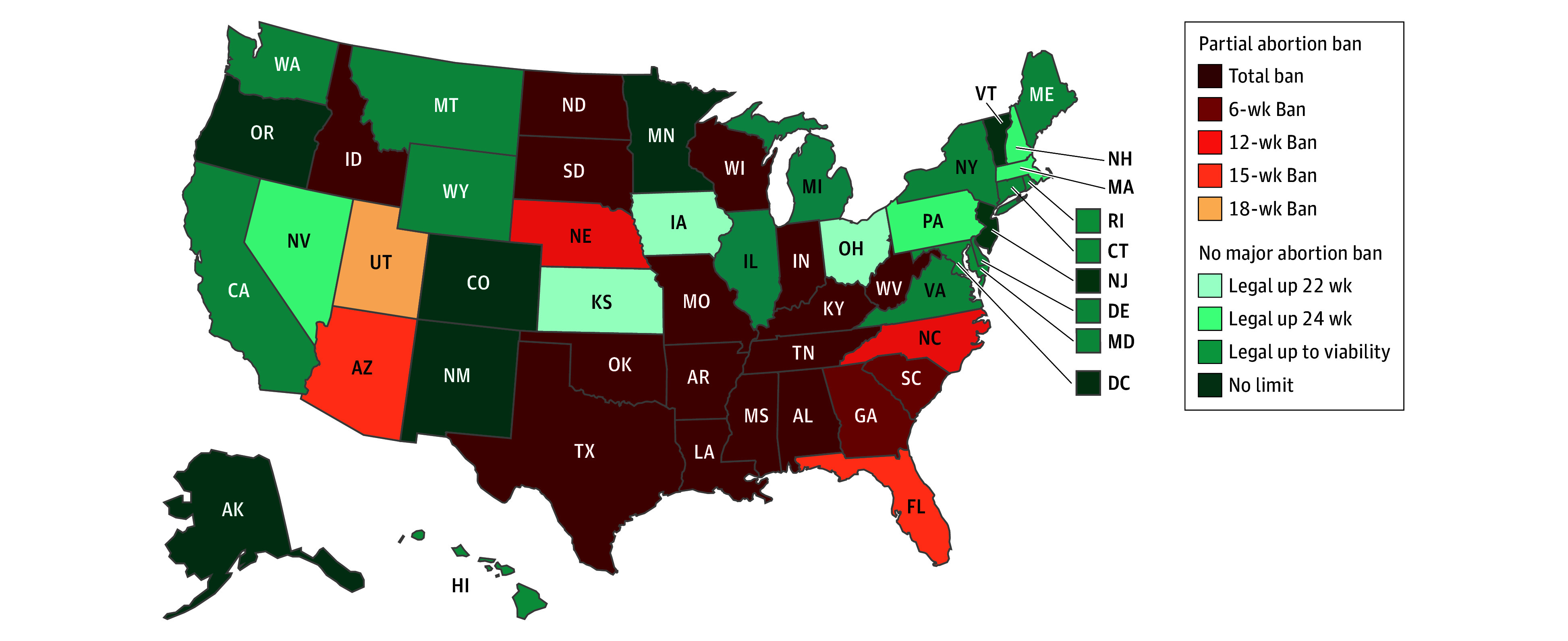
Map of State-Regulated Abortion Legality as of August 1, 2023 The legal landscape of abortion in the US near the time recruitment for this study began in September 2023. States with 2-letter postal codes are color-coded based on their abortion legality. The figure was created with MapChart.net, version 5.4.4.

Figure 1 depicts the legal landscape of abortion in the US near the time recruitment for the present study began in September 2023. The geographical clustering of these bans traces lines of race, given that the South and the Midwest have the largest proportions of Black individuals and the most total or partial abortion bans of any US region.^33^ These states are home to individuals who are disproportionately uninsured, have worse maternal and infant health outcomes, and have fewer policies aimed at expanding social services.^27,34,35^

After *Dobbs*, residents of states that restrict or ban abortion, many of whom already confront the aforementioned structural barriers, had to travel out of state or consider self-managing an abortion with medications (a medically safe but legally risky option) or other unsafe methods.^36,37^ From January through June 2023, travel for abortion doubled, with 1 in 5 patients leaving their home state for care.^38^ Nuanced patterns of travel have emerged as patients flock to “surge states,” that is, states in which abortion is legal and which attract large numbers of travelers due to geographic location.^39^ Given this precedent, understanding travel burdens post-*Dobbs* is critical to effectively support patients in restricted states.

This study examined travel to Illinois, the surge state with the largest increase in out-of-state patients in the year following *Dobbs*.^39,40^ Abortion is legal in Illinois until fetal viability, with no requirements for patient waiting periods, ultrasound scans, or parental consent for minors.^41,42^ At the time of the study, Illinois bordered 4 states with total bans, positioning it as a critical access point. We qualitatively mapped barriers and facilitators of out-of-state travelers’ journeys to abortion care in Illinois.

## Methods

This qualitative analysis assesses interview data collected at 2 Illinois clinics from September to November 2023 as part of a larger mixed-methods study.^25^ Clinic staff asked all English- and Spanish-speaking patients at least 16 years of age and medically cleared for research if they were interested in hearing about a research study, after consenting to their abortion and while waiting during standard clinical flow. The study staff (A.C., O.T., and E.J.) had no prior relationship with any participants established at the time of study commencement. The study staff described the study and assessed interest. Participants were given a study information sheet on which they provided written consent to participate in the survey (eAppendix in Supplement 1). Participants then completed a self-administered survey on a tablet, and on completion, provided verbal consent to participant in the interview. Data were collected anonymously through REDCap (Research Electronic Data Capture), a secure application for survey-based data collection.^43^ In the survey, participants self-reported their state of residence, race and ethnicity, age category, sexual orientation, and gender identity and answered a series of questions relevant to household economic stability, including household income, size, and food insecurity. Race and ethnicity data were collected to better characterize the sample. Race categories using US Census categories for guidance included African American or Black, American Indian or Alaska Native, Asian, Native Hawaiian or Other Pacific Islander, White, and other. Ethnicity categories included Hispanic or Latina/o/x or not Hispanic or Latina/o/x. Responses determined interview eligibility; individuals were eligible if they reported traveling from out of state for their abortion care, were at least 16 years of age, and could conduct an interview in either English or Spanish. Participants received $20 for completing the survey and $30 for participating in the interview. This study received review, approval, and continuous oversight from the Mass General Brigham institutional review board and followed the Consolidated Criteria for Reporting Qualitative Research (COREQ) reporting guideline.

Interviews were conducted one-on-one in a private room, audio recorded on encrypted devices, and lasted 15 to 45 minutes. The interview guide explored the abortion-seeking journey, from deciding to have an abortion to arriving at their appointment that day. The states participants traveled from were classified into 4 categories of abortion legality: total ban, partial ban, no major ban, and no limit (Figure 1), and southern vs midwestern according to the classifications of the United States Census Bureau.^44^ To capture the greatest possible demographic and geographic diversity among participants, interviews continued until all interested participants on recruitment days were included; we did not assess for thematic saturation during data collection.

### Data Analysis

We transcribed interviews verbatim and conducted directed content analysis to extrapolate quotes and map participant journeys.^45^ Coding was led by a doctoral-trained public health researcher (E.J.) and a research assistant (A.C.), who collectively conducted most interviews. Using inductive and deductive techniques, we generated a list of a priori candidate codes from the interview guide and developed additional codes throughout the process.^46^ Microsoft Excel, version 2403 (Microsoft 365) assisted in organizing thematic and content analyses. Team members (A.C., B.K.-O., O.T., and E.J.) used memos and group discussions to reveal emergent patterns. Although we did not assess for thematic saturation during recruitment due to the recruitment format, we reached saturation in the analysis with 3 commonly iterated and highly impactful factors across the study participant’s experiences. We described 4 participant journeys to care, which were chosen for their range of complexity, length of time, and diversity of demographics. To validate preliminary data interpretation, we elicited feedback from our preexisting study advisory committee, a diverse group of community members with expertise in abortion access but no role in data collection.^47^

## Results

### Participant Demographics

Of 54 out-of-state travelers offered an interview, 33 (61%) participated in the study. Most participants were between 20 and 24 years of age (n = 12 [36%]) or 30 years or older (n = 12 [36%]) and self-identified as African American or Black (n = 19 [58%]; n = 14 [42%] White). The majority of participants identified as heterosexual (n = 27 [82%]), and the entire sample identified as female or a woman (n = 33 [100%]). Most participants received public insurance (n = 18 [55%]) and lived with at least 1 child under 18 years of age in their household (n = 24 [73%]) (Table). While not collected quantitatively, in the interviews, multiple participants mentioned being the full-time caretakers for older adults or persons with disabilities in their family. A large proportion of interviewees reported financial strain, with 15 (45%) experiencing food insecurity and 10 (30%) describing the financial situation of the household as being unable to make ends meet. Although interviews were offered to be conducted by a native Spanish speaker, all participants were fluent in English, and all interviews were conducted in English.

**Table.** Study Participant Demographic Characteristics and Details of Abortion Travel to 2 Clinics in Illinois, Fall 2023

Characteristic	Participants, No. (%)
Total No.	33
Age, y	
≤19	2 (6)
20-24	12 (36)
25-29	7 (21)
≥30	12 (36)
Ethnicity	
Hispanic or Latina/o/x	6 (18)
Not Hispanic/Latina/o/x	27 (82)
Race^a^	
African American or Black	19 (58)
American Indian or Alaska Native	0
Asian	0
Native Hawaiian or Other Pacific Islander	1 (3)
White	12 (36)
Other	2 (6)
Sexual orientation	
Bisexual	3 (9)
Heterosexual or straight	27 (82)
Lesbian	1 (3)
Unsure	1 (3)
Missing	1 (3)
Gender identity	
Female or woman	33 (100)
Male, man, or transmasculine	0
Nonbinary or gender nonconforming	0
Other	0
Gravidity	
First pregnancy	6 (18)
Any prior pregnancy	27 (82)
Parity	
No prior births	13 (39)
Any prior births	20 (61)
Monthly household income levels (before taxes), $	
0-1000	9 (27)
1001-3000	17 (52)
3001-5000	4 (12)
>5000	0
Unsure	3 (9)
Participant description of money situation in their household	
Comfortable with some extras	2 (6)
Enough, but no extras	11 (33)
Have to cut back	10 (30)
Cannot make ends meet	10 (30)
Children under 18 currently living in household	
0	9 (27)
1	7 (21)
2	9 (27)
3	5 (15)
≥4	3 (9)
Experiencing food insecurity^b^	
Yes	15 (46)
No	18 (55)
Health insurance status^c^	
Public	18 (55)
Private	7 (21)
None	8 (24)
Unsure	1 (3)
Mode of payment for abortion (selected all that applied)	
Insurance	0
Charitable funding	26 (79)
Borrowed or donated funds from friend, family, or the person or man involved in pregnancy	1 (3)
Self-pay with personal funds	10 (30)
States participants traveled from (abortion legality as of September 1, 2023)	
Southern state with total abortion ban (West Virginia, Tennessee, Kentucky, Alabama, Mississippi, Arkansas, Louisiana, Oklahoma, Texas)	25 (76)
Southern state with partial abortion ban (North Carolina, South Carolina, Georgia, Florida)	2 (6)
Midwest state with total abortion ban (Indiana, Wisconsin, Missouri, North Dakota, South Dakota)	5 (15)
Midwest state with no major abortion ban (Michigan, Ohio, Illinois, Iowa, Kansas)	1 (3)
Primary mode of transport to abortion	
Car	25 (76)
Plane	8 (24)
Other (bus, train, walk, bike, and subway)	0
Mode of payment for abortion-related travel (selected all that applied)	
Charitable funding	12 (36)
Borrowed or donated funds from friend, family, or the person or man involved in pregnancy	10 (30)
Self-pay with personal funds	17 (52)
No. of days of wages lost because of the abortion appointment and related travel	
None (don’t work, it’s my day off anyway, or my time off is paid)	15 (45)
1	3 (9)
2	5 (15)
≥3	10 (30)
Time between day of deciding to have an abortion and the day of the appointment, d	
<7	9 (27)
7-14	9 (27)
15-21	4 (12)
22-28	2 (6)
≥29	9 (27)
Median (IQR)	14.0 (6.0-42.5)
Mean (SD), d	29.6 (36.5)
Completed No. of gestational weeks at abortion	
≤8	17 (52)
9-14	6 (18)
15-20	8 (25)
≥21	2 (6)

^a^ Participants self-reported all categories that applied.

^b^ Food insecurity was assessed at the end of the interview by having participants answer yes or no to the following statement: “In the past 3 months, we ran out of food and did not have enough money to buy more.” If the participant answered yes, food insecurity was identified.

^c^ Health insurance status refers to the insurance status of the participant; none of the participants were able to use insurance to pay for an abortion.

The Table also displays details related to the travel and payment for the abortion. The largest percentage of participants traveled to Illinois from a southern state with a total abortion ban (n = 25 [76%]), and most participants traveled by car (n = 25 [76%]). Although the majority of participants paid for the abortion with charitable funding (n = 26 [79%]), most travel costs were self-paid with personal funds (n = 17 [52%]). A total of 18 participants (54%) reported having lost a least 1 day of wages due to abortion related travel. From the time participants decided to terminate their pregnancy to the day of the abortion appointment, participants experienced a median of 14.0 days of delay, with a mean (SD) of 29.6 (36.5) days of delay.

### Emergent Themes: Factors Affecting Travel for Abortion Care

The Box presents a conceptual schema of common interlinking factors that shaped the travel experience of our participant pool as derived from our thematic analysis. We found that many participants described how legal restrictions constituted the most salient driver of their experiences; however, they also noted the influences of information-gathering and interpersonal support on their access to care.

Box. **Interlinking Factors Shaping Travel Experiences From out of State to Obtain Abortion Care at 2 Clinics in Illinois, Fall 2023**

**Policy landscape and abortion stigma of sending state**

Regulation of abortion access (total bans, gestational age limit, or waiting period)
Criminalization of abortion, self-managing abortion, or aiding and abetting a person receiving an abortion
Culture of judgement or support around abortion

**Information and resource gathering**

Use of search engines such as Google or AbortionFinder.org to find clinics
Referrals or information received from other health care sites or antiabortion pregnancy resource centers visited
Visibility and availability of funding for abortion and travel costs

**Interpersonal support**

Support or involvement from friends, family or the person or man involved in the pregnancy
Referrals to clinics or information about laws
Receiving assistance with transportation, childcare, financial costs, or coverage at work
Accompaniment to the clinic

The Box provides a conceptual schema of common interlinking factors that shaped the abortion travel experience of our participant pool as derived from our thematic analysis. The chart highlights the 3 primary domains of information and resource gathering, interpersonal support, and the policy landscape and stigma of the sending state. These elements interact to shape abortion seekers’ abilities to locate clinics, find social and financial support, and navigate the legal barriers to care.

#### Policy Landscape and Abortion Stigma of the Home State

The first crosscutting theme influencing travelers’ experiences was abortion laws in their home state. Participants discussed total bans, gestational age limits, waiting periods, and the criminalization of seeking or self-managing abortion or aiding another person in receiving an abortion. Many participants expressed dissatisfaction with restrictive laws in their home and reported a desire to change them.

“If I could change something about it, I would change the law. I would change the fact that you have to go hours away from home. I feel like you should be able to do it in your comfortable space in a clinic close by, rather than traveling and missing work. Because the abortion only takes about 10 minutes. It’s bizarre to come 5 hours for a 10-minute procedure.” Participant A, from a state with total ban.

Participants commonly vocalized an increased fear of discussing abortion and searching for information online since bans went into effect. The stigmatization and judgmental culture around abortions severely limited the resources they could access or share with others.

“Here, I cannot say a word. I feel like I’m going to Hell. I wish it was easier–it’s just not easy to be here.” Participant B, from a state with a total ban.

“If I could post about [abortion resources] freely without getting the hate that I know I’m going to get, I would. I wish I could share that information with a lot of people.” Participant C, from a state with a total ban.

#### Information and Resource Gathering

The second theme extrapolated was the process of information and resource gathering, which included participants planning their abortion care and related travel. Respondents described using search engines and referrals from health care professionals to locate care. Others discussed interactions with an institution outside of the mainstream health care system, such as antiabortion pregnancy resource centers, that delayed their care. Many respondents expressed difficulty locating abortion clinics and charitable funds.

“It was difficult to find [a clinic]. I tried Google to just find a place, and it wasn’t showing really anything. It was taking me to clinics where it wasn’t legal. …The place that I went to get the ultrasound first was one of the places where they were talking Christianity at me, trying to converse me out of it.” Participant D, from a state with a total ban.

“Finding which organizations have funds available is rough. It is hard. Some companies will pay for the abortion, but they won’t pay for traveling and lodging. Others won’t pay for the abortion, but they’ll pay for travel and lodging. I literally had to use a fine-tooth comb.” Participant E, from a state with a total ban.

#### Interpersonal Support

The third theme derived was the importance of interpersonal support in the abortion-seeking process. This process included help from family, friends, coworkers, partners or the other person or man involved in the pregnancy in locating clinics, organizing travel, and accompanying the individual to the clinic. Study participants expressed that having a support person accompany them to the clinic was one of the most critical aspects of their experience.

“[Having my friend here] has made it like night and day. I think that’s one of the reasons why I have been as okay as I am. I would be freaking out a lot more. It’s made all the difference.” Participant E, from a state with a total ban.

Interviewer: “What do you wish was different for anybody seeking abortion care?”

Interviewee: “Making sure everybody has support. That’s all. Because I know I’m lucky. Some people, they don’t have that support.” Participant F, from a state with a total ban.

Participants who could not arrange or afford to have a support person accompany them expressed increased levels of fear and anxiety.

“I took 2 flights coming here by myself. Whatever I’ve needed to do I’ve done it by myself, alone. And I left my baby for the first time in my life with a neighbor for 3 days just to be here. It’s a hard situation. Sometimes, you don’t have money saved to pay to have another person come with you.” Participant G, from a state with a partial abortion ban.

Frequently, the logistical and financial support a person received from their social circle determined whether they were able to make it to their appointment or not.

Interviewer: “What do you think would have happened if your support person wasn’t able to get the rental car?”

Interviewee: “I would have never came. Would have never came. I would have never made it. Never.” Participant H, from a state with a total abortion ban.

“[Childcare] was definitely the hardest part; it was finding somebody to watch her and not having to explain why.” Participant I, from a state with a total abortion ban.

### Exemplar Participant Journeys

**Figure 2.**
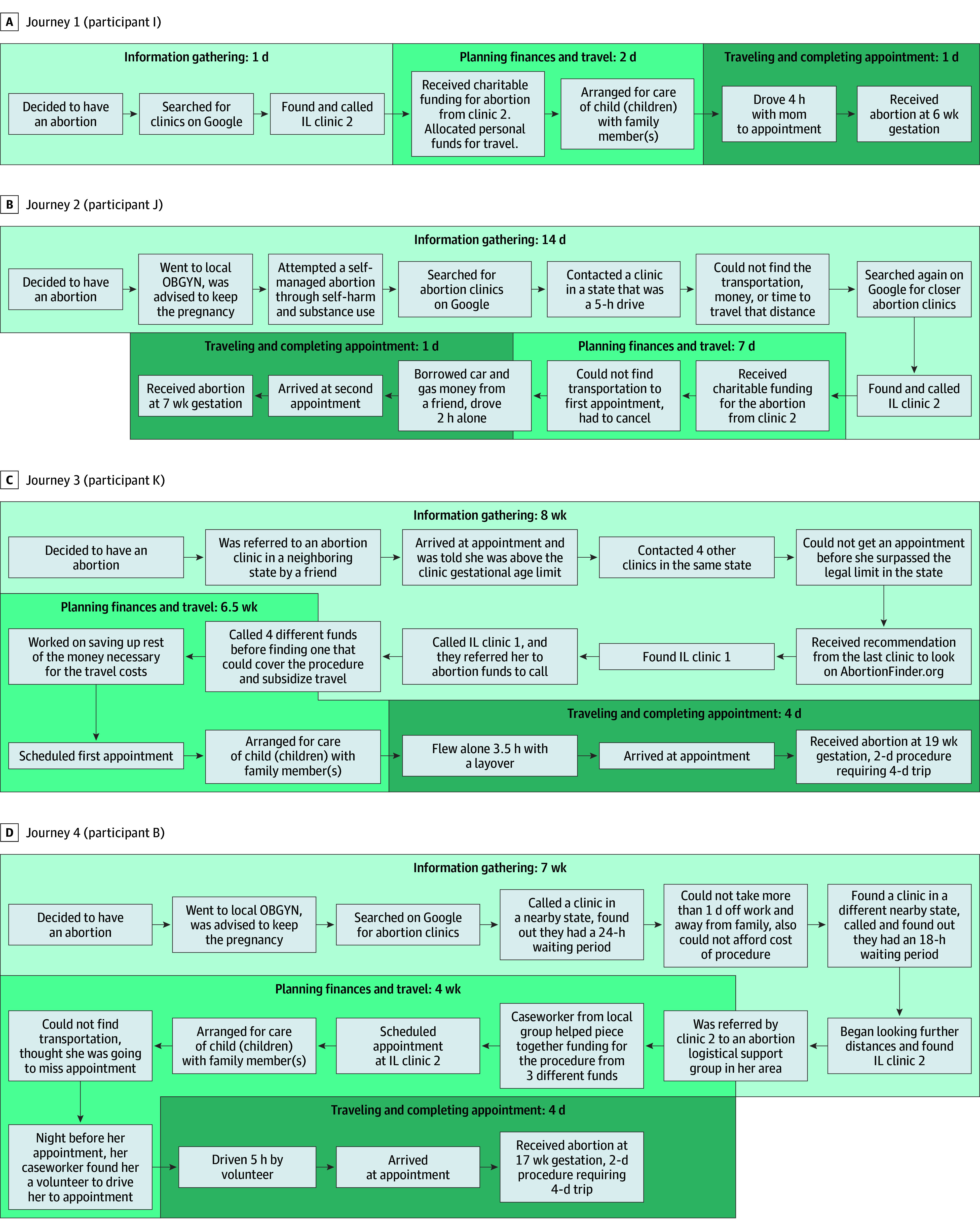
Flowcharts Depicting Participant Journeys in Searching, Planning, and Traveling Across State Lines for Abortion Care at 2 Clinics in Illinois, Fall 2023 Experiences of 4 participants are presented from the time they decided to have an abortion to the time they arrived at their appointment. Each diagram is presented in chronological order using arrows as guides. OBGYN indicates obstetrician and gynecologist.

To understand how all the interlocking factors shape individual journeys, we graphically mapped participants’ journeys to care. Figure 2 presents 4 journeys that demonstrate a range of experiences, from the less complex and time-consuming example of journey 1 to the substantial complexity and delays experienced in journeys 3 and 4. Each journey is categorized into 3 time frames: information gathering, planning travel and financial logistics, and traveling to the clinic and completing the appointment.

Journey 1 illustrates travel facilitators, including accessible information, availability of personal funds, reliable childcare, and support person accompaniment. Other participants with similarly short journeys were earlier in pregnancy, had interpersonal support or savings for travel, and lived closer to the clinics in Illinois.

Many participants expressed the greatest delays as they sought information (for participants in journeys 2, 3, and 4, these were the longest segments of their journeys). The participant in journey 2 recounted feeling hopeless and confused about whether or not she could access an abortion while living in a state where it is banned. This led her to attempt to self-manage her abortion through physical harm and substance use until she was able to find sufficient information online. Both this participant and the participant in journey 4 had reached out to their local obstetrician and gynecologist when they decided they wanted an abortion. Neither woman received adequate pregnancy options counseling nor information on how to obtain an abortion out of state. These participants expressed feeling deceived and discouraged after these encounters and were subject to even lengthier information gathering processes.

The woman in journey 3 recalled being confused and frustrated as she called multiple clinics in a nearby state before understanding she was being subjected to the same gestational age limit at each one. Once she found the Illinois clinic, she had to contact multiple abortion funds and save up her own money before being able to afford the procedure and travel costs, which made her planning finances and travel phase take 1.5 months. In journey 4, the participant sought care closer to home but was unable to do so due to waiting periods in nearby states. Once participants found a clinic and scheduled an appointment, travel was often facilitated by their social circle, such as the participant in journey 2 who was able to borrow a car and gas money from a friend. Participants in journeys 3 and 4 had 4 days of traveling and appointments to complete a surgical procedure with cervical dilation, which could have been avoided had they been able to seek care at an earlier stage in their pregnancy. Collectively, these journeys demonstrated the varied experiences of accessing abortion care across state lines and revealed charitable funding and logistical and financial interpersonal support as facilitators.

## Discussion

This qualitative exploration of travel for abortion after *Dobbs* showed nuanced challenges in information acquisition to find an abortion appointment and high levels of financial, social, and emotional stress for participants seeking abortion across state lines. Participants in our sample were disproportionately young, low-income, and in racial and ethnic minoritized groups, similar to patients seeking abortion in the US more broadly.^26,27,48^ Although there were demographic similarities across our sample, there was heterogeneity within the participants’ experiences of accessing abortion and traveling to care. To fund the abortion, most participants received charitable funding. Paying for travel varied across receiving charitable funding, borrowing or being loaned funds from someone in their social circle, and self-paying with personal funds. To travel for care, many participants lost at least 1 day of wages. Due to the extensive delays that participants encountered, they had to wait a mean of approximately 1 month (29.6 days) between deciding to get an abortion and arriving at their appointment.

These unique and extraneous journeys were shaped by 3 key elements highlighted in the Box: policy landscape and abortion stigma of the home state, information and resource gathering, and interpersonal support systems. Participants expressed that abortion bans amplified abortion stigma and stifled information on abortion access and legality. Participants’ quotes give voice to the frustration and hopelessness that abortion seekers experienced when being subjected to the legal restrictions of receiving abortion care and the social restrictions of talking about it. Participant A highlighted in her quote the desire for the law to change, allowing her to receive abortion care in her own state.

The participant journeys in Figure 2 illuminate the lack of comprehensive information on abortion access and legality in states with bans. In journeys 2 and 4, the participants believed that they were deliberately denied abortion information at their local obstetrician and gynecologist, as they were subject to biased pregnancy options counseling and were not provided with information on seeking an abortion in a legal state. The decisions among health care professionals to not refer patients to out-of-state clinics may reflect a fear of repercussions in abetting an abortion or a cultural entrenchment of antiabortion beliefs.^49,50^

When participants were able to locate an abortion clinic, finding charitable funding was another hurdle. A study of 94 abortion funds across the country found that in the first year after *Dobbs* (July 2022 to June 2023), 63% of callers received charitable support. In the second year (July 2023 to June 2024), only 54% were supported, indicating the need for financial assistance is increasingly outpacing available charitable resources in the subsequent years after *Dobbs*.^51,52^ As demonstrated by the participant journeys, available charitable funding is the only way many patients are able to access care across state lines, emphasizing a need for continued private and philanthropic investment in abortion funds. Charitable logistical assistance was also critical, as exemplified by the participant in journey 4. She may not have arrived at her abortion appointment if the volunteer from a local abortion advocacy organization had not been able to drive her. For those with a reliable social circle, logistical assistance—such as offering a car (shown in participant journey 2 and quote from participant H)—from friends and families—was also often the linchpin of a participant’s journey to care.

Accompaniment to the clinic by a support person was also regarded with tremendous value. Our data highlighted a need for charitable funds to cover the travel costs of support people, or for other investments in patient emotional comfort, such as abortion doulas, which have been shown to be associated with a more positive patient experience.^53,54^ Another key challenge was arranging child and elder care. A large majority of participants had at least 1 child living with them, and multiple respondents qualitatively noted that they were primary caretakers of an older family member. Some abortion clinics have found success in partnering with hospital day care centers and community-based childcare organizations to decrease this barrier.^55^

Every day since the *Dobbs* decision, pregnant people have undergone financially, physically, and psychologically strenuous journeys for basic health care.^56^ Overall, our findings echo prior literature on the burdens of travel for abortion during the pre*-Dobbs* era. Both before *Dobbs* and in our interviews, many individuals expressed great social, emotional, and financial stress, shame, and anxiety throughout the abortion-seeking process, which was often modulated by the abortion culture of their state and their social circle.^13,57,58,59^ Participants reported that stigma led to a reluctance to disclose the purpose and timing of their travel, which reduced the support they could receive, a finding consistent with other studies on how abortion stigma affects access.^60^

In the year after *Dobbs*, the national number of clinician-provided abortions increased.^39^ Rather than decreasing abortions, bans intentionally place a higher burden of seeking and obtaining safe health care on historically underserved communities.^25^ These groups are subjected to the financially and psychologically arduous requirements of seeking abortion out of state and the long-term effects associated with being denied an abortion, including increased household poverty, worse maternal health outcomes, and adverse effects on child development.^3,57,58,59^ To promote health and social equity, these bans should be dissolved, and federal protection for abortion should be reinstated.

Furthermore, states where abortion is legal, especially those located near restricted states, are in a critical position to enact legislation that expands and protects abortion access for those traveling from banned states. Policies such as mandated waiting periods have not been shown to have a clinical benefit, but rather increase travel burdens and logistical and financial difficulties for patients.^61^ Restrictive policies further the possibility that an individual cannot obtain a desired abortion, predisposing them to a greater chance of long-term financial stress and worse mental and physical health outcomes.^3^ To help people in the US remain healthy, abortion must be equitable and accessible to all.

### Limitations

These data should be interpreted considering several study limitations. Our data were unable to capture the experience of non-English speakers, as all interviews were conducted in English. Navigating health care institutions and planning travel presents unique challenges for non–English-speaking populations in the US, which warrants further investigation. This study was cross-sectional and involved only participants who successfully arrived at the clinics in Illinois. Thus, we cannot report on the barriers to abortion-seeking travel that are insurmountable or the negative implications that follow the denial of a desired abortion. More research is needed to identify the large-scale socioeconomic and psychological effects of having severely limited access to abortion in the southern US region. Although study enrollment was open to legal minors, few individuals under 20 years of age presented for care in these clinics during the recruitment window. Minors face unique barriers to care, such as parental involvement laws for abortion; future research should focus on understanding their experiences after *Dobbs*.^62,63^

## Conclusions

In this cross-sectional qualitative study of participants traveling across state lines seeking abortion, we found that the policy landscape and abortion stigma of the home state, information and resource availability, and interpersonal support shaped patient journeys in the post-*Dobbs* landscape. These findings can guide interventions from legislators, clinicians, abortion funds, and advocates to expand access and address systemic inequity in a post-*Dobbs* health care system.

## Notes

Supplement 1. **eAppendix.** Quality and Equity in Abortion-Seeking Travel (QuEAST Study)

Supplement 2.
Data Sharing Statement
