# Supplementary material for: Seeking Abortion Care Across State Lines After the Dobbs Decision
Source: JAMA Netw Open. 2026 Mar 9;9(3):e261068. doi: 10.1001/jamanetworkopen.2026.1068 (PMC12973109; doi:10.1001/jamanetworkopen.2026.1068)
Supplement: Supplement 1. — eAppendix. Quality and Equity in Abortion-Seeking Travel (QuEAST Study) [file jamanetwopen-e261068-s001.pdf]

# Supplementary Online Content

Cornell A, Keefe-Oates B, Thornton O, Fortin J, Gallegos A, Janiak E. Seeking abortion care across state lines after the *Dobbs* decision. *JAMA Netw Open*. 2026;9(2):e223050. doi:10.1001/jamanetworkopen.2026.3050

**eAppendix.** Quality and Equity in Abortion Seeking Travel (QuEAST Study)

This supplementary material has been provided by the authors to give readers additional information about their work.

© 2026 Cornell A et al. *JAMA Network Open*.

**eAppendix. Quality and Equity in Abortion Seeking Travel (QuEAST Study)**

**Information Sheet for Adult Participants (V4, January 2024)**

**What?** We would like to learn more about your thoughts and experiences in seeking abortion care, and how those experiences differ depending on where you are traveling from.

**Why?** The purpose of this research study is to provide information to clinical organizations, mutual aid groups, and the public about barriers to abortion access and about the ways we can help people overcome those barriers.

**Who?** Elizabeth Janiak, ScD is the person in charge of this research study. You can contact her at 617-525-9686 with any questions about the project.

**How?** You will complete a brief (10-15 minute) survey on a tablet by yourself. You may also participate in a 30-45 minute qualitative interview, which will be audio recorded. For both the survey and the interview, you will not give your name, date of birth, or any other identifying information. This study is anonymous, and we will never link any of the research data you provide to your medical record. There is no follow-up after the single survey and/or interview.

**Reimbursement?** If you choose to be part of the study, you will be given a $20.00 retail gift card for completing the survey and potentially an additional $30.00 for interview participation. You will receive the total of $20.00 or $50.00 as a single retail gift card immediately after completing the study and before you leave the clinic. We will not need to ask for, document, or save any identifying information about you in order to give you this gift card.

**Risks?** This study has very few risks. You may feel uncomfortable with questions about sensitive topics such as pregnancy characteristics and life stress. There is a risk that, if a transcript (written record) of your interview was linked to your identifying information, someone other than our study staff could become aware of your participation in this study. However, to protect against this risk we are storing all data anonymously and will never link your survey or interview data to your name or any other identifying information, even for a short time.

**Benefits?** There may be no direct benefits to taking part in the study for most participants. Some participants may benefit from having a chance to discuss their thoughts and opinions.

**Privacy?** Every effort will be made to be sure that your participation in this study, and all records about your participation, will remain private. However, privacy cannot be absolutely guaranteed. To help protect your privacy, we will never link research data to any identifying information. All information we collect will be stored only on password-protected private databases to which only our study staff will have access.

**Certificate of Confidentiality?** A federal Certificate of Confidentiality (Certificate) has been issued for this research to add special protection for information that may identify you. With a Certificate, unless you give permission, the researchers are not allowed to share your identifiable information, including for a court order or subpoena.

© 2026 Cornell A et al. *JAMA Network Open*.

Other researchers receiving your identifiable information are expected to comply with the privacy protections of the Certificate. The Certificate does not stop you from voluntarily releasing information about yourself or your participation in this study.

Even with these measures to protect your privacy, once your identifiable information is shared outside Mass General Brigham, we cannot control all the ways that others use or share it and cannot promise that it will remain completely private.

Because research is an ongoing process, we cannot give you an exact date when we will either destroy or stop using or sharing your identifiable information. Your permission to use and share your identifiable information does not expire.

The results of this research may be published in a medical book or journal, or used to teach others. However, your name or other identifiable information will not be used for these purposes without your permission.

**Problems?** If you have complaints or concerns about the research, you can call Dr. Janiak (see above). If you'd like to speak to someone not involved in this research about your rights as a research subject, or any concerns or complaints you may have about the research, contact the Partners Human Research Committee at (857) 282-1900.

**Funding?** This study is supported by grants from the Milton Fund at Harvard University, the Robert Wood Johnson Foundation, and the Society of Family Planning Research Fund.

**Statement of Consent (for participants aged 18 and older):**

I have read the information in this consent form including risks and possible benefits, and I agree to my participation in the study.

☐ I AGREE TO PARTICIPATE IN THIS RESEARCH STUDY

☐ I DO NOT AGREE TO PARTICIPATE IN THIS RESEARCH STUDY

© 2026 Cornell A et al. *JAMA Network Open*.
