# Supplementary material for: Seeking Abortion Care Across State Lines After the Dobbs Decision
Source: JAMA Netw Open. 2026 Mar 9;9(3):e261068. doi: 10.1001/jamanetworkopen.2026.1068 (PMC12973109; doi:10.1001/jamanetworkopen.2026.1068)
Supplement: Supplement 2. — Data Sharing Statement [file jamanetwopen-e261068-s002.pdf]

Data Sharing Statement

Cornell. Seeking Abortion Care Across State Lines After the Dobbs Decision. *JAMA Netw Open*. Published March 09, 2026. doi:10.1001/jamanetworkopen.2026.1068

Data
Data available: No

Additional Information
Explanation for why data not available: Due to the sensitive nature of our research topic, we will not be disclosing additional participant information beyond what is present in our manuscript. We aim to protect the anonymity and safety of participants in our study to the highest degree possible.
